# Internal Validation of a Mitochondrial DNA Control Region Sequencing Workflow Using Precision ID mtDNA Whole Genome Panel, Ion Chef™ System and Ion S5™ XL System

**DOI:** 10.3390/genes17030336

**Published:** 2026-03-18

**Authors:** Bing Hong Shue, Annabel Suan Tay, Sim Hwee Pook, See Ying Hoe, Kar Jun Loh, Christopher Kiu-Choong Syn

**Affiliations:** DNA Profiling Laboratory, Biology Division, Health Sciences Authority, 3 Biopolis Drive, Synapse, 03-14/15/16, Singapore 138623, Singapore; shue_bing_hong@hsa.gov.sg (B.H.S.);

**Keywords:** mitochondrial DNA, control region, massively parallel sequencing, Ion Torrent

## Abstract

**Background/Objectives**: Mitochondrial DNA (mtDNA) analysis is an essential tool for human identification in contexts such as disaster victim identification (DVI) and missing persons cases, where the remains may be highly degraded or even skeletonised. Traditionally, capillary electrophoresis (CE)-based Sanger sequencing has been the standard method for analysing the mtDNA control region. With the development of massively parallel sequencing (MPS) technologies, mtDNA sequencing using MPS offers advantages over traditional Sanger sequencing, such as increased sensitivity, higher throughput, and less sample consumption. The Ion Chef™ and Ion S5™ XL system from Thermo Fisher Scientific represents one such MPS system. **Methods**: We conducted an internal validation study evaluating key parameters including (a) concordance, repeatability and reproducibility; (b) potential cross-contamination; (c) sensitivity; (d) effects of library pooling on read depth; and (e) mixture sample analysis. Additionally, to mimic samples typically encountered during forensic investigations, case type samples were also used to evaluate the performance of this workflow. While the entire mitochondrial genome was sequenced in this validation study, considering that the international guidelines for full mtDNA genome analysis and interpretation have yet to be fully updated, our analysis, interpretation and subsequent implementation are limited to the control region only. **Results**: The results obtained demonstrated the reliability, sensitivity and reproducibility of this MPS workflow. **Conclusions**: This internal validation study supports the implementation of this workflow in our laboratory for the analysis of forensic casework samples.

## 1. Introduction

Mitochondrial DNA (mtDNA) analysis is a highly valuable tool for human identification in scenarios such as disaster victim identification (DVI) or missing persons cases, where the remains are highly degraded or even skeletonised. This is because mtDNA has a significantly higher copy number as compared to nuclear DNA, increasing the likelihood of successful recovery from severely compromised samples. Furthermore, mtDNA is maternally inherited and highly conserved throughout generations due to the lack of recombination, making mtDNA analysis a powerful tool for maternal lineage analysis and identification of ancient remains [1].

Traditionally, mtDNA testing is confined only to the control region using capillary electrophoresis (CE)-based Sanger sequencing as it is feasible for sequencing a short segment from a small number of samples. However, sequencing the entire mtDNA genome at high throughput using the traditional method is laborious and time-consuming. The introduction of massively parallel sequencing (MPS) technology in the mid-2000s has simplified high-throughput whole mtDNA genome sequencing with minimal human intervention and reduced sample consumption. In addition, MPS also offers higher sensitivity compared to Sanger sequencing, enabling the detection of low-level point heteroplasmies. The quantitative nature of MPS also holds potential for interpreting mixed mtDNA profiles. Given these promising benefits of MPS technology, many forensic laboratories began implementing it for mtDNA sequencing. Currently, within the forensic genetics community, the two commonly used MPS platforms are the Illumina Miseq FGx™ system, employing Illumina’s sequencing by synthesis technology, and the Ion S5™ system, based on Thermo Fisher Scientific’s Ion Torrent™ semiconductor sequencing [1,2,3,4]. While validation studies for whole mtDNA genome sequencing on both platforms have been published in the past few years [5,6,7,8,9], comprehensive analysis and interpretation guidelines for their application in forensic casework have yet to be fully updated [10]. Therefore, although our laboratory has adopted the Ion S5™ system to sequence the entire mtDNA genome, the current focus is on the analysis of the control region as the initial phase of wider MPS implementation.

In this work, we describe the internal validation study for the analysis of the mtDNA control region, conducted using the Precision ID mtDNA Whole Genome Panel, Ion Chef™ System, and Ion S5™ XL System, combined with the Converge™ software v2.3 for data analysis. The validation was carried out in accordance with the Scientific Working Group on DNA Analysis Methods (SWGDAM) Validation Guidelines for DNA Analysis Methods [11] and with reference to the developmental validation study performed for the Ion S5™ workflow [5]. The aim of this study is to evaluate the performance of this workflow, establish analysis thresholds, and develop interpretation guidelines for mtDNA control region in our laboratory.

## 2. Materials and Methods

### 2.1. Samples

This internal validation study comprised two phases. In Phase 1, we evaluated the performance of the workflow in our laboratory using known samples and negative controls. In Phase 2, we proceeded to test the workflow using samples that are commonly encountered during forensic casework.

A total of 160 sequencing reactions were performed in Phase 1 of this study (Table 1), which comprised 10 International Quality Assessment Scheme (IQAS) proficiency test samples, four control DNA samples (HLA60, 9947A, 2800M and 007), and all the negative controls. The proficiency test samples were extracted on the Maxwell^®^ FSC instrument (Promega Corporation, Seoul, Korea) using the Casework Extraction Kit and the Maxwell^®^ FSC DNA IQ™ Casework Kit sample cartridge (Promega Corporation, Madison, WI, USA) following the manufacturer’s protocol. Liquid control DNA samples were purchased from manufacturers.

Hair shafts obtained from five volunteers and six anonymised tissue samples were examined in Phase 2 of this study (Table 1).

For the hair shaft samples, 2 cm hair shafts were cleaned via mild lysis by incubating the hair fragments in 900 µL Lysis Buffer A (10 mM Tris-HCL at pH 8.0, 100 mM NaCl, 1 mM CaCl_2_, 2% SDS and 800 µg/mL proteinase K) at 56 °C for 2 h with shaking. The hair fragments were then washed three times in 800 µL Lysis Buffer B (10 mM Tris-HCL at pH 8.0, 100 mM NaCl, 1 mM CaCl_2_ and 2% SDS). The hairs were then digested in 100 µL Digestion Buffer (10 mM Tris-HCL at pH 8.0, 100 mM NaCl, 1 mM CaCl_2_, 2% SDS, 39 mM DTT and 250 µg/mL proteinase K) at 56 °C for 1 h with shaking. After digestion, the samples underwent phenol/chloroform separation and Microcon^®^ DNA Fast Flow (Millipore, Carrigtwohill, Ireland) purification to obtain the liquid DNA for sequencing [12].

For tissue samples, they were pulverised into powder, and 0.1 g of powder was extracted on the Maxwell^®^ FSC instrument using the Bone DNA Extraction Kit (Promega Corporation, Madison, WI, USA) and the Maxwell^®^ FSC DNA IQ™ Casework Kit sample cartridge following the manufacturer’s protocol.

All extracted samples were quantified on the Applied Biosystems QuantStudio™ 7 Flex Real-time PCR instrument (Thermo Fisher Scientific, Singapore) using the Quantifiler^®^ Trio DNA Quantification Kit (Thermo Fisher Scientific, Warrington, UK) prior to sequencing.

More detailed information about the samples used and sequencing runs performed can be found in Table S1.

### 2.2. Library Preparation and Quantitation

All samples were amplified with the Precision ID mtDNA Whole Genome Panel (Thermo Fisher Scientific, Pleasanton, CA, USA) according to the manufacturer’s recommended protocols [13]. For all studies except the sensitivity study, mixture study and case type sample study, 100 pg of DNA sample was used. For the case type sample study, 100 pg or the maximum volume of 15 µL was used for library preparation (Table S1). One negative control was included in each library to determine the level of background signal and to monitor for contamination throughout the entire sequencing process. All libraries were prepared on the Ion Chef™ (Thermo Fisher Scientific, Singapore) with the Precision ID DL8 Kit (Thermo Fisher Scientific, Carlsbad, CA, USA) according to the manufacturer’s recommended protocols [13]. Following library preparation, quantification of the library was carried out on the Applied Biosystems QuantStudio™ 7 Flex Real-time PCR instrument using the Ion Library TaqMan™ Quantification Kit (Thermo Fisher Scientific, Vilnius, Lithuania). Libraries were normalised to 30 pM for templating to ensure that all libraries were equally represented. Libraries that were less than 30 pM were used neat. Details on how the libraries were pooled for each sequencing run are listed in Table S1.

Two runs (Table S1, Runs 5 and 6), each consisting of 16 samples, were performed consecutively to detect any run-to-run contamination. For each run, control DNA samples (100 pg) and water were placed in alternate positions during library preparation, with the first well of the first run containing a sample and the first well of the second run containing water, to give a checkerboard pattern of control DNA and negative control. A total of 16 negative controls were obtained from these two runs.

### 2.3. Templating and Sequencing

Template preparation for pooled samples was carried out on the Ion Chef™ following the manufacturer’s recommended protocols [13]. Two 530™ chips (Thermo Fisher Scientific, Carlsbad, CA, USA) were sequenced per initialisation. Ion chips loaded with templated Ion Sphere Particles (ISPs) were placed on the Ion S5™ XL sequencer (Thermo Fisher Scientific, Singapore) for sequencing using the Ion S5™ Precision ID Chef and Sequencing Kit (Thermo Fisher Scientific, Carlsbad, CA, USA) according to the manufacturer’s recommended protocols [13].

### 2.4. Sanger Sequencing

For the case type sample study, samples were amplified with the primers listed in Table 2 [14]. A total of 1 ng or the maximum volume of 10 µL of sample DNA was used in a 25 µL PCR reaction consisting of 0.5 µM each of the forward and reverse primer and 12.5 µL of 2X NEBNext^®^ Ultra™ II Q5^®^ Master Mix (New England Biolabs, Ipswich, MA, USA). DNA was amplified using the Proflex™ PCR system (Thermo Fisher Scientific, Singapore) with the following thermal cycling conditions: initial denaturation at 96 °C for 10 min, followed by 32 cycles of 94 °C for 20 s, 56 °C for 10 s, 72 °C for 30 s, and with the final hold at 4 °C. PCR products were visually confirmed by gel electrophoresis on a 1.2% E-gel containing SYBR™ Safe (Thermo Fisher Scientific, Qiryat Shemona, Israel). The primers for control region amplification and sequencing are listed in Table 2. Primer synthesis and sequencing were conducted by the commercial laboratory “1st BASE” (Singapore).

### 2.5. Data Analysis

Primary sequencing data analysis of the mtDNA control region was performed with the Torrent Suite™ Software v5.10.0 (Thermo Fisher Scientific) and aligned to the revised Cambridge Reference Sequence (rCRS) reference genome with nucleotides 1–80 repeated after position 16,569 to allow for accurate alignment. Secondary analysis with alignment and variant calling was then performed with the HID Genotyper v2.3 plug-in with the Converge™ software v2.3.

Sequence variant frequencies were calculated by dividing the read depth of a particular nucleotide or insertions/deletions (indels) to the total read depth at that nucleotide position. For Phase 1 of this study, the following parameters were used based on the default Mito Variant Caller (MVC) thresholds: 20X for minimum total read coverage per position, 96% for confirmed variant calls, 10% for point heteroplasmy (PHP), 20% for insertions, and 30% for deletions. From the data collected in Phase 1 of this study, the thresholds for Phase 2 were modified to 200X for minimum total read coverage per position, 30% for insertions, and 40% for deletions (Table S2).

Automated variant calling was first performed by Converge™ v2.3 based on the thresholds stated above. Sequence variants not labelled as “Confirmed” were manually verified using the Integrative Genomics Viewer (IGV) function in Converge™ v2.3 by an analyst. Median read depth and strand bias (0.5 = balanced, 1 = not balanced) were calculated by Converge™ v2.3. Sequencing run performance metrics were taken from the Run Summary PDF in the Torrent Browser. Data handling and statistical analyses were performed with Excel v2512 (Microsoft Corporation).

Data analysis of Sanger sequencing results was performed using FinchTV software v1.4.0 and the mtDNAprofiler online tool developed by Yonsei DNA Profiling Group, Department of Forensic Medicine, Yonsei University College of Medicine [15].

### 2.6. Concordance Data

MPS data obtained from Phase 1 of this study were compared to the expected data published for the 14 known samples (10 proficiency test and four control DNA samples; Table S3). Haplotypes of proficiency test samples were obtained from the proficiency test provider’s expected results report. Haplotypes of the control DNA samples were obtained from Riman et al. and Churchill et al. [16,17].

## 3. Results and Discussion

In this section, we examine the performance of the sequencing runs and then evaluate the various parameters recommended by SWGDAM guidelines for internal validation: concordance, repeatability and reproducibility; contamination assessment; sensitivity; mixture sample analysis; and case type sample study [11].

### 3.1. Sequencing Run Performance Metrics

A summary of the performance metrics for all runs is included in Table S4. The manufacturer’s application guide provided a set of recommended values for various sequencing parameters to facilitate assessment of data quality before result analysis [13]. The manufacturer’s recommended values as well as an assessment of the performance metrics for all the runs are provided in Table 3. The majority of the runs met all the recommended values for the various parameters. For the runs that did not meet all the recommended values, the deviations from the recommended values were relatively small except for “Total aligned bases” in two of 12 runs.

The “Total aligned bases” for Runs 11 and 12 were much lower than the recommended 95%. According to the manufacturer’s guide, this may lead to reduced coverage for these samples during analysis. However, for these two runs, the lowest average read depth among the samples was 910X, which is still much higher than the 200X threshold set for Phase 2 of our validation. Therefore, these two runs were deemed suitable for further analysis.

### 3.2. Performance of Sequencing Workflow

To assess concordance, repeatability and reproducibility, control DNA (HL60, 9947A, 007 and 2800M) and 10 proficiency test samples were sequenced in triplicates across three different runs (Runs 1 to 3 in Table S1). Two of the runs were performed by one operator on different days, and the third run was performed by a second operator. This arrangement allowed investigation of any variability between library preparations, template preparations, sequencing runs and different operators.

Across all the replicates of the 14 known samples (n = 42), one replicate (Sample 18352) had amplicons (mt_3, mt_4 and mt_6) that fell below the 20X coverage threshold. Furthermore, the other amplicons in this replicate had unusually low read depth when compared to the other replicates. The other two replicates of this sample showed no amplicon drop-outs, and all other samples in the same sequencing run demonstrated good read depth. These observations eliminate the possibilities of any sample- or run-related issues and suggest that a pipetting error had likely occurred for this replicate during the library preparation step. Thus, this replicate was excluded from any further analysis.

#### 3.2.1. Amplicon Balance

**Figure 1:**
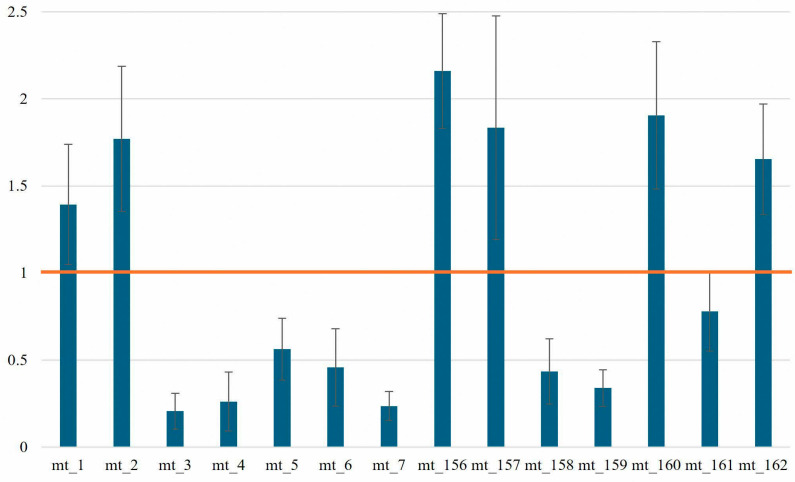
Average amplicon balance throughout the 14 amplicons covering the control region. Amplicons not depicted here are outside of the control region and not part of this study. The orange line denotes balanced amplicons.

Amplicon balance is calculated by dividing the median read depth of an amplicon with the median read depth of all amplicons. Ideally, all amplicons should have similar performance. However, previous studies have shown that an imbalance between amplicons can be observed in the Precision ID mtDNA Whole genome panel [5,17,18]. This same pattern of amplicon performance was also observed in our data, with the poorer performing amplicons coinciding with the homopolymer C stretches in the HV 1 and HV 2 regions (Figure 1). Mt_3 had the lowest amplicon balance at 0.21 (±0.10), and mt_156 had the highest amplicon balance at 2.16 (±0.33).

#### 3.2.2. Concordance, Repeatability and Reproducibility

For concordance, the haplotypes obtained from all the controls and samples were compared to the expected haplotypes (Table S3). After a manual review of the variant calls by Converge™ v2.3 using IGV, the majority (92.5%) of the variant calls were concordant to the expected haplotypes, including the two expected point heteroplasmies (PHPs). Discordant variant calls and the expected variant calls are listed in Table S5. Apart from a few discordant variant calls, which were presented as PHPs, the rest were all associated with indels, and most of them were in homopolymer regions. Homopolymer regions have always been challenging during sequencing, and it is even more so for the Ion sequencing technology, which is based on pH detection [19].

Looking at the insertion discordant variant calls, most of the issues were with calling the dominant insertion, such as the insertion at position 309 and the four-C insertion at position 573. The expected number of C insertions were detected, but they were not the major population. There were also possible false insertions at 16193C in samples where the anchor 16189T was mutated to C, resulting in a long stretch of 10 Cs. Three of the 14 known samples used in this study have this 16189C mutation.

A similar pattern was observed for deletions, where most of the discordant deletion calls were at the end of homopolymer regions, like 291del and 309del. For 291del, which is at the end of a stretch of 6 As, they were called only in Run 3. Deletions at position 291 were also observed for those affected samples in the other two runs, albeit lower than the 30% threshold. For 309del, these deletions were also observed during the developmental validation [3] and are likely false deletions given that it is supposedly a rare variant.

**Figure 2:**
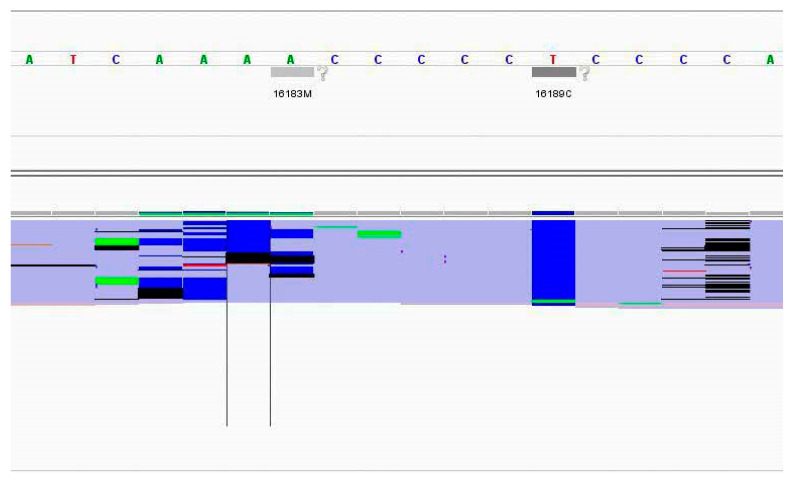
Screenshot from IGV showing an example of alignment issues at 16182M.

Lastly, there were four samples with PHP discordant variant calls. Two of the samples (Samples 17321 and 19388) had discordant calls at the A stretch before the C stretch in the HV1 region (position 16182 and 16183). These two samples have the 16189T to C mutation resulting in a long stretch of 10 Cs. This long stretch of Cs may have caused difficulty during sequencing and during alignment of reads, as shown in Figure 2, where deletions, C and A nucleotides were all simultaneously observed at positions 16182 and 16183. This resulted in the expected 16182M not being called in one of the replicates, and discordant 16182M and 16183M being called in several replicates of these two samples. For the other two samples with PHP discordant calls (Samples 20429 and 20453), 489Y was called due to C reads crossing the 10% threshold. These were only observed in Run 3 and not in the other two runs. Upon searching against the potential nuclear mtDNA (NUMT) positions provided by Li et al. [20] and checking for nuclear sequence homology using BLAST-Like Alignment Tool (BLAT), 489Y is unlikely to be a NUMT read. Since this was only observed in one of the runs and was not reproducible, it is likely to be amplification of low-level exogenous DNA.

There are two expected PHPs among the controls and samples sequenced in this study: Sample 17321 with 16182M and Sample 18352 with 64Y. For Sample 17321, 16182M was not called in one of the replicates due to alignment issues mentioned earlier, and for Sample 18352, one of the replicates yielded an incomplete profile, and therefore, no further analysis was performed. Among the two replicates left, 16182M in Sample 17321 had variant frequencies of 22.4% and 29.0%; and 64Y in Sample 18352 had variant frequencies of 42.4% and 41.2%.

#### 3.2.3. Variability of Variant Frequencies

**Figure 3:**
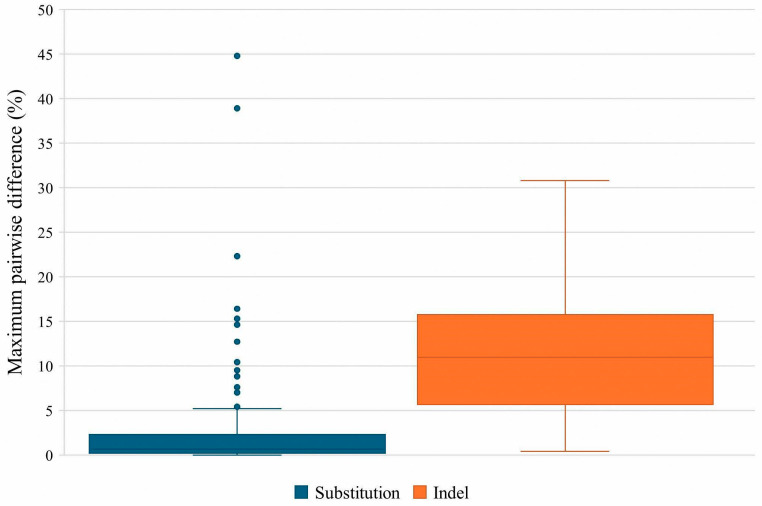
Box and Whisker plot showing the average maximum pairwise difference for substitutions and indels. Circles denote outliers observed in the data.

**Figure 4:**
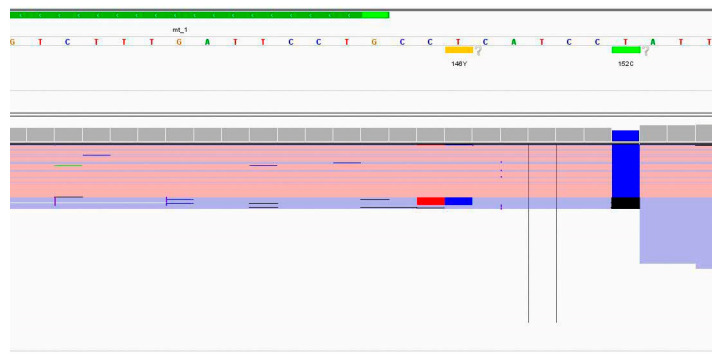
Screenshot from IGV showing an example of sequencing and/or alignment issues in Run 3.

Maximum pairwise difference in the variant frequencies of all the sequence variants (i.e., highest variant frequency minus lowest variant frequency) between the triplicates was calculated to further examine the variability between runs. The average maximum pairwise difference for substitutions was 2.9% (±6.3%) and 11.3% (±7.5%) for indels (Figure 3). The higher average and standard deviation observed for indels were expected due to the difficulty in sequencing homopolymer regions. In the developmental validation study [5], the authors obtained 0.6% (±0.9%) for substitutions and 11.6% (±9.4%) for indels. In comparison, our average maximum pairwise difference was comparable for indels but higher with respect to substitutions. This may be attributed to the outliers observed in our data. These outliers consisted of sequence variants from positions 16182 and 16183, where there is a 16189C mutation. The long stretch of Cs affected alignment in that region and thus resulted in more variable variant frequencies. There were also some outliers seen due to lower variant frequencies in certain sequence variants from Run 3. These were most likely caused by sequencing and/or alignment issues, as multiple deletions and insertions were seen in the amplicons of those variants, accompanied by extreme strand bias (>0.9) (Figure 4).

Overall, Phase 1 of our study demonstrated successful sequencing yielding haplotypes that were reproducible across the three different runs and concordant with expected results. It should be noted that caution must be taken when interpreting the HV 1 and HV 2 C-stretch regions, as there may be possible false deletions and insertions encountered, especially if mutations like 16189C are present, resulting in an extremely long C-stretch.

### 3.3. Contamination

As mtDNA analysis may be more prone to contamination, the ability to differentiate a true mtDNA variant from background and exogenous DNA is critical. This is even more so when using MPS technology, which has higher sensitivity. In this regard, a contamination study was conducted to ascertain the level of background signal present and, thereby, facilitate the identification of true variants. In this study, the presence of exogenous DNA, which could have been introduced from various sources such as equipment, consumables, reagents, operator and even the laboratory environment, was assessed. In addition to the 16 negative controls from Runs 5 and 6, 10 negative controls collected from all the other studies in this performance verification were also included in the contamination assessment.

**Figure 5:**
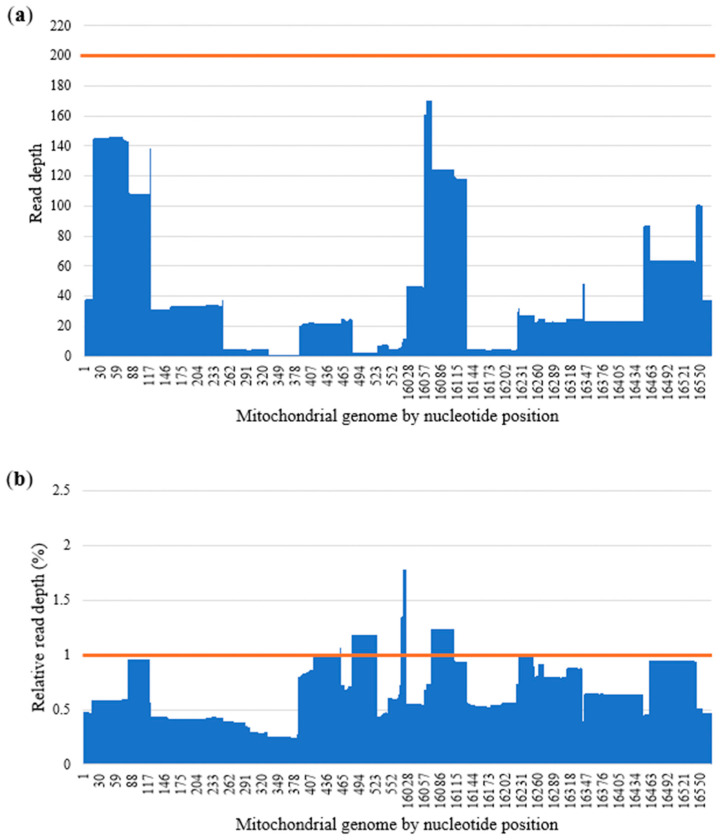
(**a**) Average read depth across the control region for the 26 negative controls included in this study. The orange line denotes 200X read depth. (**b**) Average relative read depth across the control region for the 22 negative controls included in this study. The orange line denotes 1% relative read depth.

The average read depth of all 26 negative controls was calculated and found to range from 0.6X (±0.9X) to 170.5X (±176.8X) across the control region of the mtDNA genome (Figure 5a). As such, to eliminate false variant calls from low-level exogenous DNA, a read depth of 200X, which is above the highest observed average read depth of 170.5X, was used as the minimum total read coverage threshold in Phase 2 of this study. Of the 26 negative controls, 22 negative controls had an accompanying positive control in the same run with DNA input at 100 pg. This allowed the evaluation of the relative read depth, which was calculated by dividing the read depth at each nucleotide position of the negative control over the read depth at the same nucleotide position of a positive control in the same run. The average relative read depth for the 22 negative controls ranged from 0.2% (±0.4%) to 1.7% (±3.4%) across the control region of the mtDNA genome; importantly, the majority (88.3%) of the control region had an average relative read depth of less than 1% (Figure 5b).

From the data collected, background mtDNA levels were generally below 200X coverage, with a relative read depth that was considerably lower than the default PHP threshold of 10%. While there were reads exceeding 200X coverage detected in some of the negative controls, this was rare—among the 26 negative controls, each with 14 amplicons, only 12 out of 364 (3.3%) total amplicons exceeded 200X coverage. However, when analysing samples with low coverage, such background noise might manifest as PHP, and care must be taken when interpreting such PHPs.

#### Bioinformatic Negatives

An advantage of MPS technology over traditional Sanger sequencing is its higher throughput, made possible with multiplexing. During library preparation, to aid in sample identification, each sample in the library is uniquely labelled with a barcode. As these barcodes are made up of short sequences of nucleotides, they could also be a potential source of contamination. There is a risk of carry-over from previous library runs, and with the pooling step, there is also a risk of other samples being picked up by the software during analysis. As part of the contamination study, 32 bioinformatic negatives were evaluated. Two of the bioinformatic negatives produced an error during analysis by Converge™ v2.3 and were excluded from further analysis. Data from the remaining 30 bioinformatic negatives were analysed for 16 barcodes not associated with any sample during library preparation (Table S1, Runs 5 and 6), to detect exogenous barcode contamination and carry-over between runs.

**Figure 6:**
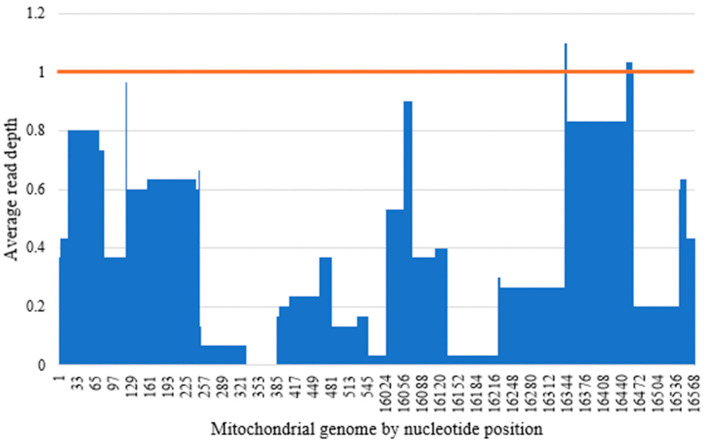
Average read depth across the control region for the 30 bioinformatic negative controls included in this study. The orange line denotes 1X read depth.

The average read depth ranged from 0X (±0X) to 1.1X (±3.6X) across the control region of the mtDNA genome (Figure 6), with a maximum of 19X observed. This maximum of 19X observed is lower than the 20X minimum read depth threshold used in this study. Across the control region, 98.7% of the nucleotide positions had an average read depth of less than 1X.

### 3.4. Sensitivity Study

Sensitivity of the Precision ID mtDNA Whole Genome Panel was determined by sequencing a range of serially diluted DNA extracted from two different samples (Run 4 in Table S1). The aim of this sensitivity study was to determine the quantity of input DNA at which drop-out of amplicons or variant calls would occur. Each of the two samples was subjected to two-fold dilutions six times to yield seven serially diluted input DNA (150 pg, 75 pg, 37.5 pg, 18.8 pg, 9.4 pg, 4.7 pg and 2.3 pg). The results from this sensitivity study are summarised in Table 4.

#### 3.4.1. Read Depth

**Figure 7:**
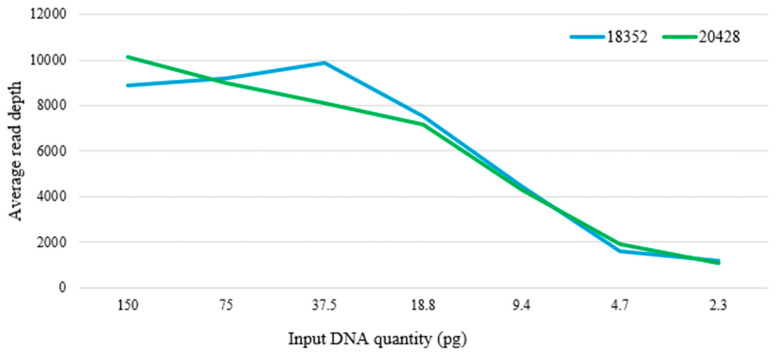
Average read depth across the control region for the two samples at different quantities of input DNA.

The effects that different quantities of input DNA have on read depth were determined by calculating the average read depth of each nucleotide across the control region of the mtDNA. The general trend observed is as expected—as DNA quantities decreased, the average read depth across the control region declined (Figure 7).

Based on the default read depth threshold of 20X, coverage of the entire mtDNA control region could be achieved for all input DNA amounts for sample 20428. However, for sample 18352, drop-out of amplicons was observed at 37.5 pg, 9.4 pg, 4.7 pg and 2.3 pg. Using IGV to view the sequence data, the drop-outs were all found to be at amplicon mt_3. With the sudden drop in read depth and a mutation (242T) at the primer-binding site for mt_3, these observations indicated that the amplicon drop-outs were likely due to a reduction in amplification efficiency caused by a primer-binding site mutation. When using 200X coverage as a cut-off value and disregarding the mt_3 drop-out for sample 18352, both samples encountered drop-outs at 4.7 pg and 2.3 pg.

In the contamination study, the highest average relative read depth of the negative controls against positive controls with input DNA at the recommended 100 pg was 1.7%. In contrast, the average relative read depth of the negative controls against samples at varying input DNA amounts was higher, ranging from 0.42% to 8.21% (Table 4). From this data, the default PHP threshold of 10% would be effective in eliminating false PHPs caused by exogenous DNA, even in low DNA samples.

#### 3.4.2. Concordance

Regarding the samples’ haplotypes, the majority of the variants were concordant with the expected variants. This included the PHP in sample 18352, which was correctly identified across all input DNA amounts. However, at 4.7 pg and 2.3 pg for sample 18352, multiple false PHPs were observed. These PHPs were likely due to the stochastic variation at low read depth, stemming from the difficulty in discerning true variants from exogenous DNA and/or NUMTs. This was also supported by the increasing trend observed in the average relative read depth of negative controls in these sequencing runs. In sample 20428, an additional 248R PHP was observed for input DNA amounts ranging from 150 pg to 9.4 pg. Upon verification of the sequence data using IGV, the PHP was assessed to be a low-level PHP hovering at approximately 10% variant frequency. For 4.7 pg and 2.3 pg, the PHP fell below the 10% threshold and was, therefore, not called. The data for sample 20428 in the concordance study was revisited, and this 248R PHP was also observed in the sequence data but was not called by Converge™ v2.3. This phenomenon was also reported in the developmental validation study and may be attributed to the difficulty in alignment for that region due to a deletion at position 249 in the small overlap between mt_2 and mt_3 [5]. As this PHP is at about 10% variant frequency and adjacent to a deletion, it is possible that it was not picked up during the generation of the expected haplotype for the proficiency test, as most laboratories still relied on Sanger sequencing for mtDNA analysis.

#### 3.4.3. Effect of Sample Pooling on Read Depth

**Figure 8:**
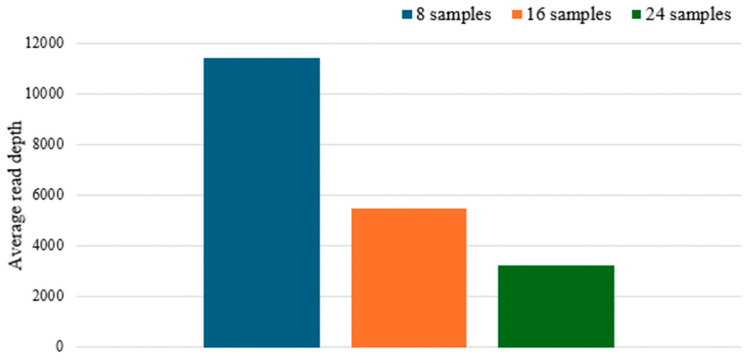
Average read depth across the control region when 8, 16 or 24 samples were sequenced per chip.

In addition to sensitivity, we also studied how pooling a different number of libraries onto a single sequencing chip affects average read depth (Runs 7, 8 and 9 in Table S1). As expected, the average read depth of the control region decreased as the number of samples loaded per chip increased (Figure 8). The average read depth roughly halved when the number of pooled samples increased from 8 to 16 and from 16 to 24. It should be noted that forensic casework samples may consist of low quantity and/or low quality (i.e., degraded) DNA. As such, the number of samples loaded per chip can influence the likelihood of obtaining a full haplotype of the control region. A possible recommendation would be to pool up to 24 samples for pristine good quality reference samples while limiting the pooling for casework samples to only 16 or less.

### 3.5. Mixtures

A mixture study was conducted with two different mixture sets, with the aim of illustrating and understanding the characteristics of mixed samples sequenced using MPS (Run 10 in Table S1). For each mixture set, samples of known haplotypes were mixed at a nuclear DNA ratio of 20:1, 10:1, 5:1, 2:1, 1:2, 1:5 and 1:10. To obtain the mixture ratios, the input DNA quantity of one contributor was maintained at 2 pg, while the input DNA quantity of the second contributor varied from 40 pg to 0.2 pg. The mixtures’ input DNA amount ranged from 42 pg (20:1 ratio) to 2.2 pg (1:10 ratio). Despite the low input DNA, all the mixtures reached the 20X read depth threshold at all the nucleotide positions across the control region of the mtDNA genome.

**Figure 9:**
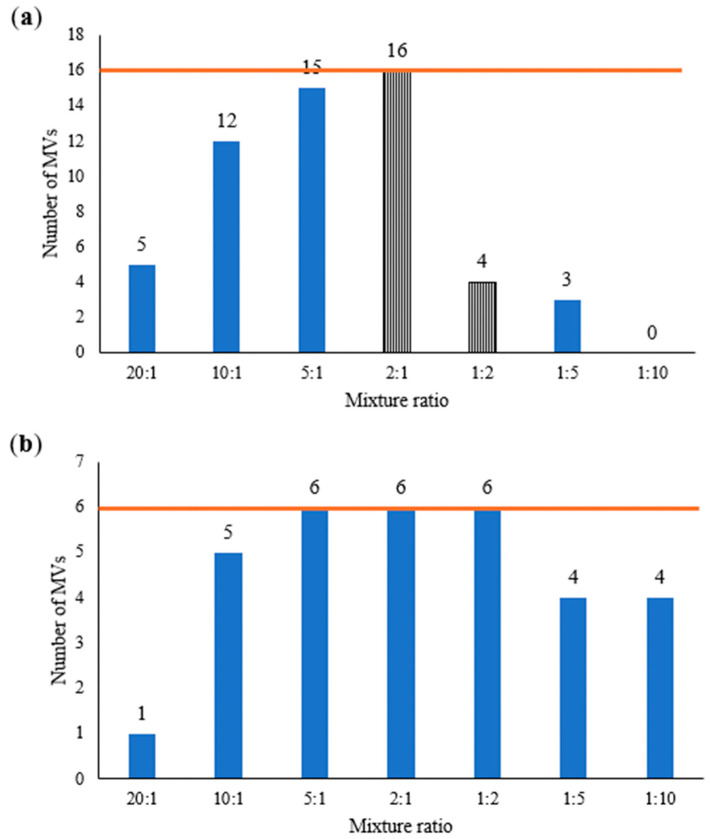
Number of MVs for each mixture ratio for (**a**) Mixture 1 and (**b**) Mixture 2. As the haplotypes of the contributors are known, the expected number of MVs is indicated on each graph by the orange line. Mixtures containing MVs that are not part of either contributor’s mtDNA sequence are indicated by striped bars.

The presence of a mixture can be identified by the number of positions with two or more nucleotides observed (mixed variant point sites or MVs). Indels were not included in the total number of MVs due to their high variability. The number of MVs for each mixture ratio for the two mixture sets is shown in Figure 9.

With a 10% PHP threshold, the maximum number of PHPs expected for an individual should be no more than two [21]. Using the criterion of three or more MVs to indicate the presence of a mixture, “mixtures” could be identified in all the samples except mixture ratio 1:10 in Mixture 1 and 20:1 in Mixture 2.

The more similar the input DNA amount of each contributor (i.e., 2:1 and 1:2 ratios), the more likely it is to observe variants from both contributors and, hence, the more MVs observed. The number of MVs peaked at mixture ratio 2:1 for Mixture 1 and at mixture ratios 5:1, 2:1 and 1:2 for Mixture 2. For both Mixtures 1 and 2, the number of MVs did not follow a symmetrical bell curve pattern, which is what would have been expected from such a mixture ratio series. This is likely due to the use of nuclear DNA quantification as a proxy for mtDNA input amount during the preparation of the mixture ratio series, which would not reflect the true ratio of mtDNA from each contributor [22]. In addition, two unexpected MVs that did not belong to the two known contributors could be seen at the 2:1 mixture ratio and in the 1:2 mixture ratio of Mixture 1, as indicated by the striped bars in Figure 9a. This could be the result of stochastic effects as well as the reduced ability to differentiate between true mtDNA variation, NUMTs and exogenous DNA at lower DNA input amounts [5].

Besides using MVs to recognise mixtures, the distribution of variant frequencies can also be used. From our data, 57.14% of the called variants in this mixture study showed a variant frequency of less than 96%. This contrasted with the data from the single contributor samples used in the concordance study, where only 11.28% of the called variants showed a variant frequency of less than 96%. For the 20:1 mixture in Mixture 2, although there was only one MV detected, the variant frequencies of a few other called variants were observed to be slightly lower than 96%. Even though they are not above the PHP threshold of 10%, they suggested the possibility of a low-level minor contributor present. Hence, a general trend of lower variant frequencies throughout the sample variant calls may be a useful indicator of a mixture. Table 5 shows the average variant frequencies across all variants for each mixture. The more similar the input DNA amount of each contributor, the lower the average variant frequency.

Although MPS has unlocked the possibility of interpreting mtDNA mixtures, it remains a challenge, especially when there is stochastic variation resulting in additional MVs that are brought about by low DNA input amounts. From this study, mixtures can be identified by evaluating both the number of MVs and the distribution of variant frequencies. The presence of three or more MVs as well as a general trend of variant frequencies < 96% throughout the sample are good indicators of the presence of a mixture.

### 3.6. Case Type Samples

The objective of this study was to evaluate the entire sequencing workflow on forensic samples that would typically require mtDNA sequencing, such as blood, hair shafts and human skeletal remains. As blood samples (proficiency test samples) had already been evaluated in Phase 1 of this study, Phase 2 focused on the more challenging hair shaft and bone tissue samples. Furthermore, from the data collected in Phase 1, the thresholds were revised for use in Phase 2 (Table S2). A total of five hair shaft and six bone tissue samples were sequenced in Phase 2 via both Sanger sequencing and the Ion S5™ XL workflow (Runs 11 and 12 in Table S1). A summary of the results is shown in Table 6, and the haplotypes of the samples are shown in Table S6. All samples were sequenced in duplicates using the Ion S5™ XL workflow except for Case 1-1, Case 1-3 and Case 1-4, as there were insufficient DNA extracts for two sequencing runs.

For Sanger sequencing, the results obtained for “Hair 3”, “Case 1-1” (HV1), “Case 1-3” (HV1) and “Case 1-4” (HV1) were of poor quality and not usable. Consequently, concordance to the sequencing results for “Hair 3” could not be determined. Out of the 11 samples, only two hair shaft samples achieved full mtDNA sequences in both HV1 and HV2 regions. For Ion S5™ XL sequencing, full mtDNA control region sequences were obtained for nine out of 11 samples. The average read depth ranged from 910X (±556X) to 14,715X (±11,106X) across the 11 samples. For the two samples with partial sequences obtained (“Case 1-3” and “Case 1-4”), they both had the same amplicon drop-out. This was likely due to a primer-binding site mutation resulting in a sudden decrease in read depth for that amplicon. A similar effect was observed for samples “Case 1-1” and “Case 1-2”, although both still achieved a read depth of more than 200X for that amplicon. Although there was drop-out of a single amplicon, the Ion S5™ XL sequencing of these two samples (“Case 1-3” and “Case 1-4”) still yielded more information with a larger sequencing range as compared to Sanger sequencing. Furthermore, for “Case 1-1”, “Case 1-3” and “Case 1-4”, no autosomal short tandem repeat (STR) profile was obtained when amplified using the Globalfiler™ kit. In such situations, Ion S5™ XL sequencing proves valuable towards recovering useful mtDNA control region information for human identification purposes.

Comparing the 122 variants detected using Ion S5™ XL sequencing to the 66 variants detected using Sanger sequencing for the 11 case type samples, only two of the variants were discordant. Both discordant variants were in sample “Hair 1”. 309del was detected using Ion S5™ XL but was not seen in Sanger sequencing. This was likely a false deletion, as this discrepancy was also observed multiple times in our earlier concordance study. The other discordant variant was a point heteroplasmy 16304Y detected using Ion S5™ XL, while a 16304C was detected using Sanger sequencing. Upon reviewing the Sanger sequencing results for “Hair 1”, a low-level T was seen in the electropherogram. This low-level point heteroplasmy was more readily observed when using Ion S5™ XL technology due to greater sensitivity.

In addition to comparing to Sanger sequencing data, the mtDNA haplotypes obtained in this study were also compared to data obtained from sequencing reference samples. Blood samples from the same volunteers who provided the hair shafts had been previously sequenced using Ion S5™ XL during initial testing of the instrumentation. There were two discordant variants observed between the five pairs of hair and blood samples. One of the discordant variants was a 309del observed in the blood sample of the volunteer who contributed “Hair 2”, which was likely a false deletion. The other discordant variant was the same 16304Y point heteroplasmy observed in “Hair 1”, but a 16304C was detected in the corresponding blood sample. This is not unusual given that tissue-specific mutations can occur, and the rate of point heteroplasmy has been found to be higher in hair (35%) as compared to blood (18%) [23].

### 3.7. Implementations

From our validation study, we determined our minimum total read coverage threshold to be 200X, which is a value that is above the highest average read depth of the negative controls in our contamination assessment. We have also adopted the value of 10% as our PHP threshold by looking at the average relative read depth of negative controls against both positive controls and samples at different input DNA amounts. Both thresholds will aid in eliminating the majority of the spurious variant calls arising from background exogenous DNA.

Additionally, from the discordant variant calls identified in our concordance study, the majority of them are indels around homopolymeric C regions at positions 302 to 310, 568 to 573 and 16184 to 16193. As such, those indels at these regions should not be used for direct forensic comparisons, which is in agreement with international forensic guidelines [10].

Furthermore, our mixture study demonstrated that the presence of three or more MVs and/or a general trend of variant frequencies < 96% throughout the sample indicate that the mtDNA profile obtained is likely a mixture.

In addition, when interpreting low-level mtDNA profiles, analysts may consider re-sequencing, if possible, to confirm PHP variants.

## 4. Conclusions

In this internal validation study, we evaluated the performance of mtDNA sequencing using the Precision ID mtDNA Whole Genome Panel with the Ion Chef™ and Ion S5™ XL systems. Expected haplotypes were obtained for all the known samples in our concordance study, and some potential sequencing artifacts involving indels were identified. The contamination study demonstrated that there was a low-level baseline read depth generally lower than 200X for negative controls. In the sensitivity study, stochastic variations were observed, which may have resulted from exogenous DNA and/or NUMTs, and can manifest as false PHPs appearing in samples with low-level input DNA. When sequencing case type samples such as bone and hair, our workflow was able to obtain the complete mtDNA control region haplotype.

The present study demonstrated that our MPS sequencing workflow of the mtDNA control region can produce reliable and reproducible results. As a first step of our MPS implementation, our validation and implementation are focused only on the analysis and interpretation of the mtDNA control region, and further studies to expand to the whole mtDNA genome may be conducted in the future.

## Figures and Tables

**Table 1:** Summary of experiments done for Phase 1 and 2.

Phase	Study	Sample Details	Run
1	Concordance, Reproducibility and Repeatability	14 samples in triplicates	1 to 3
	Contamination	16 blanks alternating with control samples	5 and 6
	Sensitivity-Dilution series	2 samples at 7 different input DNA amounts	4
	Sensitivity-Sample pooling	Reuse libraries prepared from Runs 1 and 2	7 to 9
	Mixture	2 sets of 2-person mixtures at 7 different mixture ratios	10
2	Case type samples	5 hair samples in duplicates; 6 tissue samples (3 in duplicates)	11 and 12

**Table 2:** Primers used for Sanger sequencing.

Primer Name	Sequence
PCR primers for HV1	
F15971	TTA ACT CCA CCA TTA GCA CC
R16410	GAG GAT GGT GGT CAA GGG AC
PCR primers for HV2	
F15	CAC CCT ATT AAC CAC TCA CG
R484	TGA GAT TAG TAG TAT GGG AG
Sequencing primers for HV1	
F15971	TTA ACT CCA CCA TTA GCA CC
R16410	GAG GAT GGT GGT CAA GGG AC
Sequencing primers for HV2	
F15	CAC CCT ATT AAC CAC TCA CG
R408	CTG TTA AAA GTG CAT ACC GCC

**Table 3:** Manufacturer’s recommended values and an assessment of all the runs in this study.

Parameters	Recommended Values	Comments
ISP loading	>50%	All runs passed
Key signal	>60	All runs passed
Enrichment	≥98%	All runs passed
Total usable reads	~30–40%	All runs > 30% except Run 6 (29%)
Polyclonal	25–50%	All runs passed
Test fragment	1%	All runs ~ 1% (0.9–1.4%)
Adapter dimer	≤1%	All runs passed except Runs 2 (1.6%), 4 (1.7%), 5 (2.5%) and 10 (3.5%)
Low quality	≤50%	All runs passed
Histogram	Similar pattern to recommended	All runs performed similarly (mean and median read length ranged from 122 bp to 138 bp)
Total aligned bases	≥95%	All runs passed except Runs 11 (89%) and 12 (71%)

**Table 4:** Results of the sensitivity study showing average read depth, percentage of the control region that reached the 20X and 200X read depth threshold, average relative read depth of the negative control and presence of stochastic variation.

Sample	Nuclear DNA Amount (pg)	Average Read Depth (X)	Percent of Control Region > 20X	Percent of Control Region > 200X	Average Relative Read Depth of Neg (%)	Stochastic Variation
18352	150	8866	100.00	95.54	1.38	
	75	9199	100.00	95.54	1.15	
	37.5	9846	95.63	95.54	1.17	
	18.8	7532	100.00	95.54	1.45	
	9.4	4451	95.54	95.54	2.74	
	4.7	1620	95.54	91.18	8.21	Yes
	2.3	1206	95.54	84.58	7.20	Yes
20428	150	10127	100.00	100.00	0.42	
	75	8994	100.00	100.00	0.47	
	37.5	8119	100.00	100.00	0.45	
	18.8	7148	100.00	100.00	0.50	
	9.4	4330	100.00	100.00	0.96	
	4.7	1898	100.00	82.09	1.89	
	2.3	1062	100.00	82.09	4.06	

**Table 5:** Average variant frequencies for each mixture ratio in the two mixture sets.

Mixture	Mixture Ratio	Average Variant Frequency (s.d.)
1	20:1	92.45% (8.97%)
	10:1	90.39% (6.21%)
	5:1	77.60% (13.55%)
	2:1	71.08% (17.26%)
	1:2	94.75% (7.31%)
	1:5	96.19% (4.46%)
	1:10	98.59% (2.38%)
2	20:1	95.00% (4.52%)
	10:1	88.16% (6.72%)
	5:1	82.40% (8.74%)
	2:1	76.43% (10.77%)
	1:2	77.76% (10.90%)
	1:5	88.06% (6.82%)
	1:10	91.67% (5.09%)

**Table 6:** Summary of results obtained from sequencing hair shaft and bone tissue samples. Control DNA sample 007 was also sequenced as a positive control.

Sample	Sanger		Ion S5™ XL	Concordance
	HV1 (16024–16365)	HV2 (73–340)	Full Control Region (1–576, 16024–16569)	
“Hair 1”	Partial	Full	Full	No
“Hair 2”	Full	Full	Full	Yes
“Hair 3”	No usable data	No usable data	Full	-
“Hair 4”	Partial	Partial	Full	Yes
“Hair 5”	Full	Full	Full	Yes
“Case 1-1” (Femur)	No usable data	Full	Full	Yes
“Case 1-2” (Femur)	Partial	Full	Full	Yes
“Case 1-3” (Spine)	No usable data	Full	Partial	Yes
“Case 1-4” (Spine)	No usable data	Full	Partial	Yes
“Case 2-1” (Femur)	Partial	Full	Full	Yes
“Case 2-2” (Tooth)	Partial	Full	Full	Yes
“007”	Full	Full	Full	Yes

## Data Availability

Raw data is unavailable due to privacy, legal or ethical restrictions.

## Supplementary Materials

The following supporting information can be downloaded at: https://www.mdpi.com/article/10.3390/genes17030336/s1. Table S1: Summary of sequencing runs performed; Table S2: Input parameters for HID Genotyper plugin; Table S3: Expected haplotypes for the samples and control DNAs used for this study; Table S4: Summary of sequencing runs’ performance metrics; Table S5: Discrepancies in variant calls (yellow for deletions, green for insertions and blue for PHP) when compared to the expected haplotype; Table S6: Haplotypes obtained from the case type sample study.
